# Differential Micronuclei Induction in Human Lymphocyte Cultures by Imidacloprid in the Presence of Potassium Nitrate

**DOI:** 10.1100/tsw.2010.9

**Published:** 2010-01-08

**Authors:** Polychronis Stivaktakis, Dimitris Vlastos, Evangelos Giannakopoulos, Demetrious P. Matthopoulos

**Affiliations:** Department of Environmental and Natural Resources Management, University of Ioannina, Agrinio, Greece

**Keywords:** genotoxicity, micronuclei (MN), pesticides, imidacloprid, potassium nitrate (KNO3), square wave cathodic stripping voltammetry (SW-CSV)

## Abstract

Humans are exposed to pesticides as a consequence of their application in farming or their persistence in a variety of media, including food, water, air, soil, plants, animals, and smoke. The interaction of pesticides with environmental factors may result in the alteration of their physicochemical properties. Square wave cathodic stripping voltammetry (SW-CSV), a technique that simulates electrodynamically the cellular membrane, is used to investigate whether the presence of potassium nitrate (KNO_3_) in the culture medium interferes with the genotoxic behavior of imidacloprid. The cytokinesis block micronuclei (CBMN) method is used to evaluate imidacloprid's genotoxicity in the absence or presence of KNO_3_ in the culture medium and, as a consequence, its adsorption by lymphocytes. Comparing micronuclei (MN) frequencies in control and imidacloprid-treated blood cell cultures, statistically significant differences were not detected. KNO_3_ did not induce MN frequencies compared to control. Statistically significant differences in MN frequencies were observed when blood cell cultures were treated with imidacloprid in the presence of increasing concentrations of KNO_3_. SW-CSV revealed that by increasing KNO_3_ molarity, imidacloprid's concentration in the culture medium decreased in parallel. This finding indicates that imidacloprid is adsorbed by cellular membranes. The present study suggests a novel role of a harmless environmental factor, such as KNO_3_, on the genotoxic behavior of a pesticide, such as imidacloprid. KNO_3_ rendered imidacloprid permeable to lymphocytes, resulting in elevated MN frequencies.

## INTRODUCTION

The human diet is characterized by the presence of numerous naturally occurring chemicals in trace amounts. All these chemicals, constituents of processed and nonprocessed food, have been mainly considered as harmless to human health. Among the chemicals, potassium nitrate ($KNO_3$) is found as a preservative in processed food[1] and inorganic nitrate ($NO_3^-$) is found in large quantities in green, leafy vegetables[2]. A Ploughman's lunch contains approximately 2 m*M* of $NO_3^-$[3], while nitrite and nitrate levels in human circulation were calculated to be between 4 and 81 μ*M*, which may represent dietary intake differences[4]. In the meantime, a healthy diet may contain 0.5–2 m*M* of $NO_3^-$[5]. Thus, $KNO_3$ is classified by the Environmental Protection Agency (EPA) as a class III toxicity agent with limited pesticidal use[6].

Humans are exposed to a large number of industrially made chemicals, designed for various purposes, among which are those to be used for crop protection. Several of these chemicals are responsible for serious negative side effects, such as biochemical malfunctions or genetic instability[7,8]. These chemicals, in order to reveal their side effects, require initially to be adsorbed by the cells. According to their possible side effects, they are characterized as harmless, mutagenic, carcinogenic, or toxic. In order to exert their activity, they have to enter living systems, otherwise failing to do so, they are not able to exert their activity and are usually considered as harmless.

Pesticides constitute a heterogeneous class of chemicals representing an important group of environmental pollutants. In Europe, more than 3 million tons are released into the environment yearly[9]. This widespread use of pesticides, and the long and persistent exposure of farmers to them, as well as their contaminants in food, render the mutagenic and carcinogenic risk assessment a public health concern.

Imidacloprid, a systemic chloronicotinyl insecticide with soil, seed, and foliar uses, blocks the nicotinergic neuronal pathway that is more abundant in insects than in warm-blooded animals. It is a General Use Pesticide (GUP) classified by the EPA as a class II and class III toxicity agent[10]. Imidacloprid was found to induce DNA damage in a dose-related effect in earthworms[11], as well as to increase the frequency of DNA adducts in pesticide-treated calf thymus cells, indicating pesticide-induced genotoxicity[12].

The genotoxic and mutagenic activities of certain pesticides have been studied both in *in vitro* and *in vivo* systems using cytogenetic end points, such as the cytokinesis block micronuclei (CBMN) method[13,14,15]. The CBMN method in human lymphocytes, developed by Fenech and Morley[16], uses cytochalasin-B, an inhibitor of actin polymerization that prevents cytokinesis, while it permits nuclear division[17,18]. As a result, binucleated (BN) cells are produced, which are scored for the presence of MN[18,19,20].

A rather important technique to simulate electrodynamically the adsorptive ability of cellular membranes is square wave cathodic stripping voltammetry (SW-CSV), using a hanging mercury drop electrode (HMDE). The HMDE is a capillary electrode with a mercury drop at its edge. The mercury drop acts as an electrode surface. It has been widely used in environmental and biological samplings. Its function is based on the application of a fixed voltage between 200 and –1200 mV, targeted at the redox of the electroactive species, such as imidacloprid, which has been adsorbed on its surface. Electrons flowing between the adsorbed electroactive species and the electrode surface are expressed in current (measured in nanoamperes) that corresponds with these species in solution. Electroactive species move towards the electrode surface by diffusion, while they are adsorbed via electrostatic interactions[21,22]. Via stripping voltammetry (SV), the adsorption at the HMDE of dithiocarbamate pesticides[23], mitomycin-C[24], and imidacloprid[25] was studied.

The present work focuses on the synergistic action between an environmental factor, such as $KNO_3$, and a pesticide, such as imidacloprid. The possible side effects of $KNO_3$ on imidacloprid genotoxicity towards cultured human lymphocytes were investigated, using both the CBMN method and the SW-CSV.

## MATERIALS AND METHODS

### Reagents

Ultrapure Milli-Q water, Millipore Academic system, was used throughout the experiments. Imidacloprid (RDH 37894; purity: 99.9% by HPLC) obtained from Sigma-Aldrich and $KNO_3$ (GA 18517; purity: 99.9% by HPLC) obtained from Fluka were used without further purification.

Imidacloprid stock solution (100 μ*M* = 25.6 ppm) was prepared by dissolving 1.18 mg of insecticide in 46.07 ml of Milli-Q water and stored in the dark at 4°C. This imidacloprid concentration is well below its solubility limit[26], while it corresponds to the mean value of previously reported concentrations[11,27,28]. Standard solutions were prepared daily from the stock solution by dilution to the appropriate concentration.

The buffer solution used for voltammetric measurements was Britton-Robinson, prepared from a stock solution containing 10 m*M* phosphoric acid, 10 m*M* boric acid, and 10 m*M* acetic acid adjusted to the desired pH value, with NaOH. This buffer system is commonly used in analogous voltammetry experiments[29] because of its ability to stabilize pH in a broad range (i.e., pH 4–10) and to act as a supporting electrolyte.

## Apparatus and Software

For the voltammetric measurements of imidacloprid, an electrochemical analyzer (Model TraceLab50, Radiometer Analytical) was used to control the voltage of a three-electrode system. As a working electrode, a Radiometer HMDE was used with a drop area of 3 $mm^2$ controlled by pneumatic connection with nitrogen (99.999%) at p = 1 bar. The reference electrode (Ag/AgCl-KCl = 3 M), type TR020, and auxiliary platinum electrode, type TM020, for POL150 were also from Radiometer. This system was connected with a Pentium II PC.

All voltammetric measurements were carried out at 2°C. As shown previously, low temperature facilitates the adsorption of imidacloprid on the mercury drop surface[25]; therefore, a considerable sensitivity improvement can be achieved at 2°C.

The voltammetric experiments were carried out with a water-thermostated double-wall electrochemical cell (type CP021) from Radiometer Analytical. The temperature of the electrochemical cell was controlled by water circulation via a thermostated circulator operating in the temperature range of 1–50°C. The temperature was constantly monitored *in situ* in the measuring cell, with a digital thermometer inserted through a sealed opening at the top cap of the measuring cell. Before each measurement, the system was temperature equilibrated for at least 30 min. This protocol was found to provide stable temperatures within ±0.3°C[23,25].

## Instrumental Parameters

The SW-CSV technique was used with:

- *Cell parameters* — Electrode HMDE, stirrer at 525 rpm, purge time 600 sec, accumulation time ($t_{acc}$) 60 sec, waiting time 10 sec, Hg-drop growth time 0.7 sec
- *Signal parameters* — Accumulation potential ($E_{acc}$) = –1060 mV[25], step duration 0.04 sec, step amplitude 1 mV, pulse amplitude +50 mV
- *Current range* — Minimum 10 nA, maximum 10 mA, under our experimental conditions the detection limit for imidacloprid was 0.25 μ*M*. The pH measurements were carried out with a CRISON GLP21 pH-meter.

## Micronucleus Test in Human Lymphocytes *In Vitro*

Blood samples were obtained from two healthy nonsmokers, without previous known contact with pesticides. Donors aged between 20 and 25 years.

Whole blood (0.5 ml) was added to 6.5 ml Ham's F-10 medium (Invitrogen), 1.5 ml fetal calf serum (Invitrogen), and 0.3 ml phytohemagglutinin (Invitrogen) to stimulate cell division. The appropriate chemicals were added 41 h postculture initiation to final imidacloprid concentration of 20 μ*M*, and of $KNO_3$ to final concentrations of 2, 10, and 20 μ*M* in separate treatments. In mixed treatments, the final concentrations were 20 μ*M* for imidacloprid plus 5, 10, 15, and 20 μ*M* for $KNO_3$. Mitomycin-C (Sigma) at final concentration of 1.5 μ*M* served as positive control. Cultures were incubated at 37°C for 72 h. Three hours after the addition of the chemicals, 6 μg/ml cytochalasin-B (Sigma) was added at 44 h postculture initiation. Cells collected by centrifugation at 72 h postculture initiation, fixed with freshly made methanol/acetic acid (Riedel-de Haen/Merck) mixture (3:1 v/v) after mild hypotonic treatment, were stained with Giemsa (Fluka)[15,28]. At least 1000 BN cells with preserved cytoplasm were scored per slide, for each donor and for each case, in order to calculate the frequency of MN. Standard criteria[19] were used for scoring MN.

The Cytokinesis Block Proliferation Index (CBPI), given by the equation: $CBPI = M_1 + 2M_2 + 3(M_3 + M_4)/N$, where $M_1$, $M_2$, $M_3$, and $M_4$ correspond to the numbers of cells with one, two, three, and four nuclei and N is the total number of cells[30], was calculated by counting at least 2000 cells in order to determine possible cytotoxic effects.

### Analytic Determination of Imidacloprid from Human Lymphocyte Cultures

One milliliter of supernatant, derived from 72-h blood cultures in the presence of imidacloprid, was mixed with 9 ml of absolute ethanol[31]. The mixture was centrifuged at 1500 rpm for 10 min and 4 ml of the resulting supernatant were mixed with 6 ml of 10 m*M* Britton-Robinson buffer and transferred in the stripping voltammetry cell. Quantitative analysis was performed based on a calibration curve.

### Statistical Analysis

Statistical analysis of MN data was made by the G-test for independence on 2x2 tables. This test is based on the general assumption of the $\chi^2$ analysis, but offers theoretical and computational advantages[32]. The G-test was evaluated using the data analysis statistical software Minitab.

## RESULTS

### SW-CSV

Fig. 1 shows a square wave (SW) voltammogram for 20 μ*M* imidacloprid incubated in human lymphocyte cultures, in the presence of two concentrations of $KNO_3$ — 5 μ*M* (solid line) and 20 μ*M* (dotted line), respectively.

The overall signal in Fig. 1 is typical for imidacloprid molecules adsorbed on the HMDE. Control experiments (data not presented) demonstrated that the SW signal intensity decrease is not due to $KNO_3$. $KNO_3$ presence in the buffering system of the SW voltammogram builds an ionic atmosphere around the HMDE, resulting in the reduction of the electroactive specie of imidacloprid. Under these conditions, imidacloprid is adsorbed onto the surface of the HMDE. This observation is common in voltammetry[33]. Apparently $KNO_3$ reduces the negative potential of lymphocyte membranes, rendering lymphocytes permeable to imidacloprid. The decrease in signal observed in the presence of 20 μ*M* $KNO_3$ (dotted line) is due to imidacloprid being adsorbed by lymphocytes. The decreased signal can be observed more easily if current intensity is correlated to $KNO_3$ concentration (see Fig. 2).

Fig. 2 depicts the polarographic current intensity vs. $KNO_3$ concentration in lymphocyte cultures. A decrease of current intensity from 415 to 363 nA is observed when $KNO_3$ concentration is increased from 0 to 20 μ*M*.

The effect of adsorbed imidacloprid on cultured cells was further investigated by the CBMN method.

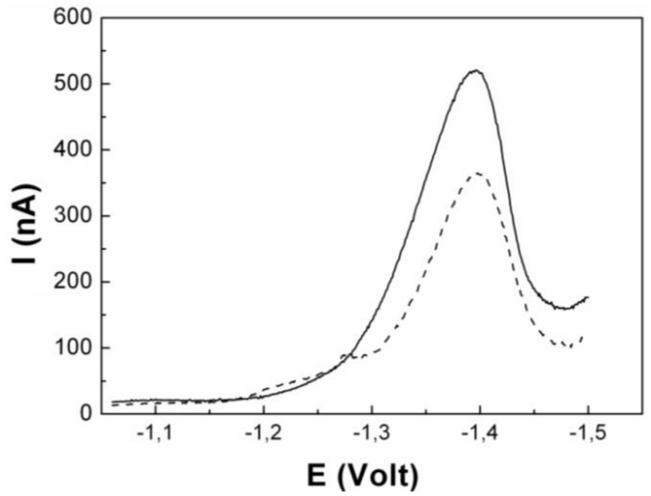

**FIGURE 1.** Stripping voltammogram of 20 μ*M* imidacloprid in lymphocyte culture media, for two different incubation concentrations — (solid line) 5 μ*M* and (dotted line) 20 μ*M* of $KNO_3$. Experimental conditions: pH = 7.4, T = 2°C, $E_{acc}$ = –1060 mV, $t_{acc}$ = 80 sec in 10 m*M* Britton-Robinson buffer.

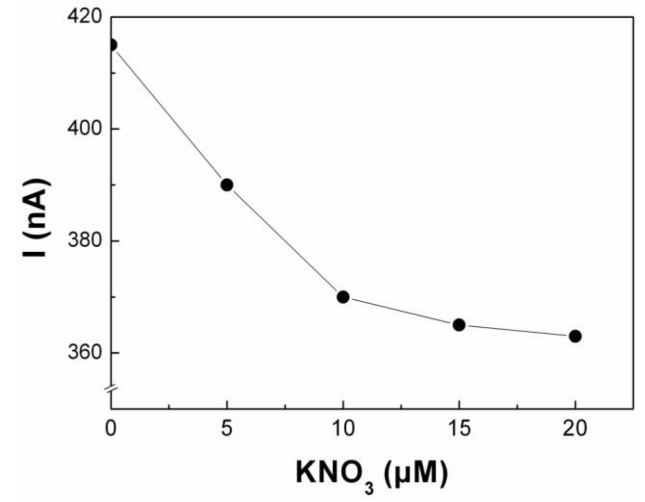

**FIGURE 2.** Effect of $KNO_3$ concentrations on the SW current intensity peak for 20 μ*M* imidacloprid in lymphocyte cultures. Blood samples were incubated at 37°C in the presence of imidacloprid.

## Micronucleus Test in Human Lymphocytes *In Vitro*

Table 1 depicts the results obtained from peripheral blood lymphocyte cultures treated with imidacloprid and $KNO_3$, separately or in mixtures.

Imidacloprid and $KNO_3$ separate treatments were not able to induce statistically significant increases in the frequency of MN and binucleated micronucleated (BNMN) cells. In the case of mixed treatments, imidacloprid + $KNO_3$, an over twofold increase of MN and BNMN frequencies, was observed in the concentrations of 20 + 15 and 20 + 20 μ*M*, respectively, compared to the control. A statistically significant increase in relation to control cultures was evaluated in BNMN frequencies from the concentration of 20 + 15 μ*M* and in the MN frequencies from the concentration of 20 + 10 μ*M* (see Table 1).

**Table 1.**

Frequencies of micronucleated binucleated cells (BNMN) and micronuclei (MN), induced in human lymphocyte cultures in separate or mixed *in vitro* imidacloprid and $KNO_3$ treatments.

| Concentration (μM) | BN cells[a] scored | BNMN[b] MF(‰)±se[e] | MN[c] MF(‰)±se[e] | CBPI[d] MF(‰)±se[e] |
|---|---|---|---|---|
| Imidacloprid | | | | |
| 0 | 2000 | 5.5±0.5 | 6.5±0.5 | 2.00±0.09 |
| 20 | 2000 | 7.0±0.0 | 8.0±1.0 | 2.03±0.08 |
| $KNO_3$ | | | | |
| 2 | 2000 | 5.0±1.0 | 5.0±1.0 | 2.21±0.03 |
| 10 | 2000 | 5.0±0.0 | 5.5±0.5 | 2.22±0.08 |
| 20 | 2000 | 5.0±0.0 | 6.0±1.0 | 2.19±0.07 |
| Imidacloprid + $KNO_3$ (mixture) | | | | |
| 20+5 | 2000 | 10.5±0.5 | 10.5±0.5 | 2.03±0.03 |
| 20+10 | 2000 | 10.5±0.5 | 11.0±1.0* | 2.00±0.06 |
| 20+15 | 2000 | 15.0±0.0** | 15.5±0.5** | 1.97±0.03 |
| 20+20 | 2000 | 15.5±0.5** | 16.5±0.5** | 2.12±0.01 |
| Mit-C (positive control) | | | | |
| 1.5 | 2000 | 78.5±6.5*** | 84.0±6.0*** | 1.37±0.16 |

[a] binucleated cells; [b] micronucleated binucleated cells; [c] micronuclei; [d] Cytokinesis Block Proliferation Index; [e] mean frequencies (‰) ± standard error, * p<0.05, ** p<0.01, *** p<0.0001 (G-test)

Our data of 20 + 5 combined mixtures indicated marginally no significant statistical differences in both BNMN and MN frequencies ($p$ = 0.057) compared to the control value. In the meantime, the same data appear to have a possible inducible effect, as their absolute values are almost twofold increased from their corresponding control values. A similar condition was observed at the concentration of 20 + 10 μ*M* with regard to BNMN frequencies.

The imidacloprid and $KNO_3$ cytotoxic effect was evaluated by the determination of CBPI. Regarding the cytotoxic index in all tested cytogenetic end points (see Table 1), no differences were observed between control and imidacloprid- and/or $KNO_3$-treated cultures.

The reported control and positive control frequencies of MN were consistent to the literature, and authenticate our experimental procedure and treatment observations[28,34,35].

The presence of $KNO_3$ at concentrations higher than 10 μ*M* in combination with 20 μ*M* imidacloprid in culture media induces a statistically significant increase of MN frequencies ($p$ < 0.05 and 0.01, respectively) compared to control, while in separate treatments no statistical differences are observed, as seen in Fig. 3.

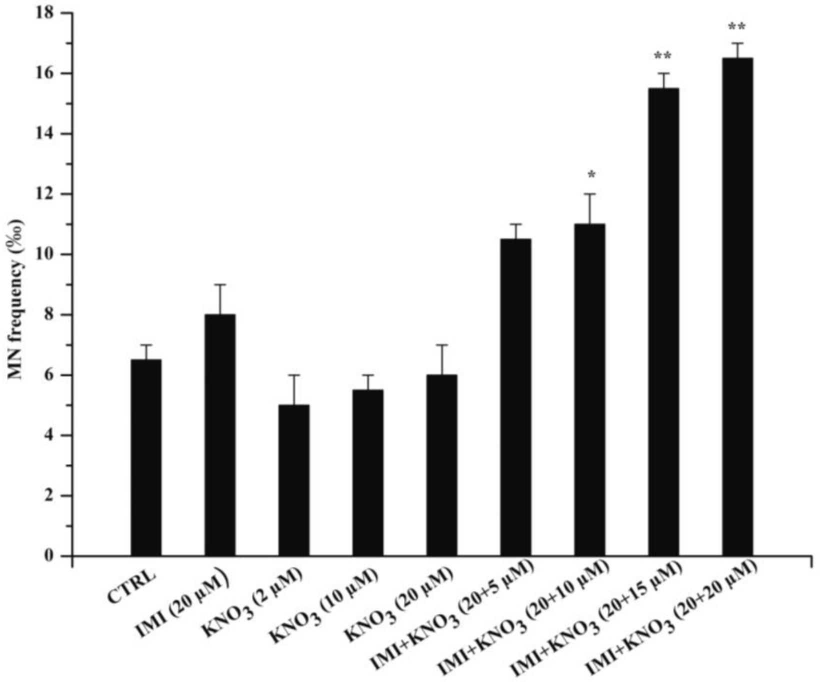

**FIGURE 3.** Effects of imidacloprid (IMI) and $KNO_3$ as pure compounds, and as mixtures on MN frequency (mean ± standard error) in cytokinesis-blocked human peripheral lymphocytes. * = $p < 0.05$, ** = $p < 0.01$ (significant differences between control [CTRL] and treated cultures by G-test).

## DISCUSSION

Skepticism has gradually developed over studying the synergistic action of pesticides and their genotoxic side effects to humans and other organisms. Many variables intervene in the study system, rendering it too complex, not easily reproducible, and increasing the workload exponentially. In addition, scientists agree that the synergistic effects of agricultural chemical mixtures on human health are controversial and further studies on this issue are needed[36].

The present study poses an additional speculation on studying the synergistic effects between pesticides and environmental factors. For this purpose, the CBMN method was used in parallel to the SW-SCV electroanalytical technique.

There is a relationship between polarographic and/or voltammetric behavior and genotoxic properties of organic compounds. By studying the mechanisms of their electrode reactions, useful clues can be provided to elucidate the mechanism of their interaction with living cells and their fate in the environment[22].

SW-CSV was used to study the effect of $KNO_3$, a "harmless" chemical, on imidacloprid's genotoxicity to human lymphocyte cultures *in vitro*. This analytical voltammetric technique simulates electrodynamically the cellular membrane by applying a certain potential to a HMDE. By this system, the adsorptive ability of the cellular membrane towards imidacloprid, in the presence or absence of $KNO_3$, could be evaluated.

The CBMN method was used to evaluate imidacloprid genotoxicity in the absence or presence of $KNO_3$.

Neonicotinoids, including imidacloprid, are the most important new class of insecticides of the past 3 decades. They are increasingly replacing the organophosphates and methylcarbamates. Neonicotinoids have low activity on mammals relative to insect nicotinic acetylcholine receptors, providing a mechanistic basis for their safety[37].

Controversial results regarding imidacloprid's genotoxic effect to cultured human lymphocytes were previously reported[27,28]. Imidacloprid was found to induce statistically significant differences of MN frequencies in cultured human peripheral blood lymphocytes after 0.1 and 0.5 μg/ml dose treatments[27], while *in vitro* treatments of 0.1–100 μg/ml did not result in any significant change in the frequency of MN in the same cytogenetic end point[28]. The per thousand (‰) of MN values at 0.1 and 0.5 μg/ml of imidacloprid (approximately 4 and 6.5, respectively[27]) were in accordance to the data reported by Demsia et al.[28]; however, control value differences were reported (approximately 2.5[27] vs. 4.5[28]), which may explain their statistical analysis differences. Meanwhile, administering 100 and 200 mg/kg b.w. imidacloprid in rats did not result in any significant change *in vivo*. However, the frequency of MN in rat polychromatic erythrocytes was statistically elevated at the concentration of 300 mg/kg b.w.[28].

The negative *in vitro* genotoxic result in our findings from the separate treatment of 20 μ*M* imidacloprid and the absence of imidacloprid's genotoxic effect in separate treatments on human lymphocytes *in vitro* at concentrations up to 400 μ*M*, as well as the weak genotoxic effect on rat bone marrow *in vivo*[28], indicate a relative safety of this pesticide upon human exposure. Costa et al. recently reported that imidacloprid at concentrations lower than 20 μ*M* is not genotoxic to human lymphocytes *in vitro*, in the presence or absence of S9 metabolic activation. However, a significant increase in MN frequency was noticed at 20 μ*M* of imidacloprid with metabolic activation, but it is not clear whether the effect is due to imidacloprid or its metabolites[38].

A gradual reduction of imidacloprid's SW signal in culture medium with increasing $KNO_3$ concentration was observed by voltammetry. This is an indication that imidacloprid interacting with $KNO_3$ is adsorbed by lymphocytes. In the absence of $KNO_3$, this reduction is not observed, while the SW signal is not altered by the presence of $KNO_3$ (see Figs. 1 and 2).

The calculated data from the electroanalytical method and genotoxicity experiments using the combination of imidacloprid and $KNO_3$ in cultured human lymphocytes denote the possibility of imidacloprid becoming genotoxic upon its interaction with a "harmless" chemical such as $KNO_3$ (see Fig. 3). It should be referred that the concentrations of $KNO_3$ used in the present study are below the levels of nitrite and nitrate reported in the human diet[4,5].

Our present data indicate that the combination of "harmless" environmental factors with pesticides that have not been classified as genotoxic to humans may interact, resulting in the alteration of the pesticides' physicochemical properties. Such alteration may convert harmless-to-humans pesticides into harmful ones with unidentified side effects. The latter could be an indication of reconsidering our classification of natural or man-made chemicals as harmless or potentially harmful ones.

Thus, further studies, including genotoxic, electroanalytical, physicochemical, and molecular assessment, are required before drawing final conclusions on the separate and/or combined with environmental factors action of pesticides.

## ACKNOWLEDGMENTS

We thank Assoc. Prof. Y. Deligiannakis for collaboration on the SW measurements.
